# Development of a yeast heterologous expression cassette based on the promoter and terminator elements of the *Eremothecium cymbalariae* translational elongation factor 1α (*EcTEF1*) gene

**DOI:** 10.1007/s13205-018-1224-0

**Published:** 2018-03-29

**Authors:** Tomas Linder

**Affiliations:** Department of Molecular Sciences, Swedish University of Agricultural Sciences, Box 7015, 750 07 Uppsala, Sweden

**Keywords:** Heterologous expression, Hygromycin, Selection marker, Yeast

## Abstract

A new expression cassette (*EC0*) consisting of the fused 5′ and 3′ intergenic regions (IGRs) of the *Eremothecium cymbalariae* translational elongation factor 1α (*EcTEF1*) gene was evaluated through expression of the bacterial hygromycin B phosphotransferase (*hph*) resistance gene in the common baker’s yeast *Saccharomyces cerevisiae*. Progressively shorter versions of the *hph*-containing *EC* cassette (*hphEC1* though *hphEC6*) with trimmed 5′ and 3′ *EcTEF1* IGRs were tested for their ability to confer resistance to hygromycin B in *S. cerevisiae*. Hygromycin B resistance was retained in all six generated *hphEC* variants up to a concentration of 400 mg/L. The *hphEC6* cassette was the shortest cassette to be assayed in this study with 366 and 155 bp of the *EcTEF1* 5′ and 3′ IGRs, respectively. When tested for deletion of the *S. cerevisiae* proline oxidase gene *PUT1*, the *hphEC6* cassette was shown to successfully act as a selection marker on hygromycin B-containing medium. The *hphEC6* cassette could be placed immediately adjacent to a *kanMX4* G418 disulfate resistance marker without any discernable effect on the ability of the yeast to grow in the presence of both hygromycin B and G418 disulfate. Co-cultivation experiments under non-selective conditions demonstrated that a *PUT1* deletion strain carrying the *hphEC6* cassette displayed equivalent fitness to an otherwise isogenic *PUT1* deletion strain carrying the *kanMX4* cassette.

**Electronic supplementary material:**

The online version of this article (10.1007/s13205-018-1224-0) contains supplementary material, which is available to authorized users.

## Introduction

During the past 4 decades, the genetic engineering of yeasts and other microorganisms has become a multi-billion dollar industry. The common baker’s yeast *Saccharomyces cerevisiae* is the most commonly used yeast systems in synthetic biology applications although other species of budding yeasts (phylum *Ascomycota*, sub-phylum *Saccharomycotina*) are gaining in popularity. The traditional approaches to genetic engineering in yeast require condition-specific selection marker genes for the isolation of successful transformants. The design of dominant selection markers such as antibiotic resistance genes tends to employ promoter sequences from constitutively active and highly transcribed “house-keeping genes” such as those involved in protein synthesis, central carbon metabolism, or cell cycle progression. Strong constitutive promoters may also be desirable for some synthetic biology applications such as the rewiring of metabolic pathways or increasing pathway flux.

Translational elongation factor 1α (Tef1) is one of the most abundant proteins in actively dividing yeast cells (Ghaemmaghami et al. 2003) and the strong activity of the *TEF1* gene promoter has lead to the development of a number of expression cassettes based on the *TEF1* intergenic regions (IGRs) from yeasts (Müller et al. 1998; Ahn et al. 2007). The 5′ and 3′ IGRs of the *TEF1* gene from the filamentous yeast *Eremothecium gossypii* (syn. *Ashbya gossypii*) has become one of the most widely used expression cassettes (commonly designated “*MX*”) for strong, constitutive expression of dominant selection markers in the genetic engineering of yeasts (Steiner and Philippsen 1994; Wach et al. 1994). The *MX* cassette has been shown to work for the expression of resistance genes in a wide spectrum of yeast species including the fission yeast *Schizosaccharomyces pombe* (Bähler et al. 1998) despite its very distant evolutionary relationship to budding yeasts (Beimforde et al. 2014).

However, the ubiquity of the *MX* expression cassette in the design of dominant selection markers for the genetic engineering of yeasts often results in transgenic strains that are modified several times with multiple *MX* cassettes carrying different resistance genes. The repeated use of the *MX* cassette or any other sequence element within genomic integration constructs will progressively decrease targeting efficiency of subsequent transformations within the same yeast genome (Davidson and Schiestl 2000). Accumulation of integrated sequence elements that contain longer stretches of identical sequence also introduces a risk of undesirable chromosomal rearrangements during genetic crosses and may introduce artifacts in high-throughput genetic analyses such as synthetic genetic arrays. Repeated use of a single or a limited sub-set of expression cassettes can also be problematic in synthetic biology applications such as heterologous expression of multigene biosynthetic pathways, where tandem arrays containing identical regulatory sequences can result in recombination-mediated excision of transgenes.

In the case of selection markers, one approach to circumvent the accumulation of identical regulatory elements within the genome of a yeast strain has been to remove the selection marker post-integration through heterologous expression of a recombinase such as Cre (Güldener et al. 1996). This approach has the added advantage of enabling repeated use of the same selection marker and thereby selection strategy. However, repeated recombinase-mediated selection marker recycling in *S. cerevisiae* has been shown to cause significant chromosomal rearrangements and gene loss (Solis-Escalante et al. 2015). An alternative approach is to introduce expression cassettes containing new promoter and terminator elements. For instance, there have been variants of the *MX* cassette described where the *EgTEF1* terminator sequence have been substituted for that of other genes such as the *S. cerevisiae ADH1* and *CYC1* genes (Janke et al. 2004). Although this reduces the risk of unwanted recombination events with any pre-existing *MX* cassettes within the yeast genome, it also introduces a new risk of recombination events with the endogenous terminator elements of the *ADH1* and *CYC1* genes if these cassettes were to be used in the transformation of *S. cerevisiae*.

Newly sequenced yeast genomes are valuable resource for new regulatory sequences for heterologous expression of selection markers and other transgenes. As IGRs are non-coding, they evolve faster than coding gene sequences while retaining their regulatory function in related species. Thus, a promoter or terminator element can be transplanted into the genome of a more distantly related species and function, even though the overall sequence homology between promoters may be beyond detection. The present study sought to develop a new constitutive expression cassette based on the IGRs of the *TEF1* gene from the filamentous yeast *Eremothecium cymbalariae*, which is the closest relative of *E. gossypii* with a sequenced genome at the time of writing (Wendland and Walther 2011).

## Methods

### Yeast integration constructs

**Fig. 1:**
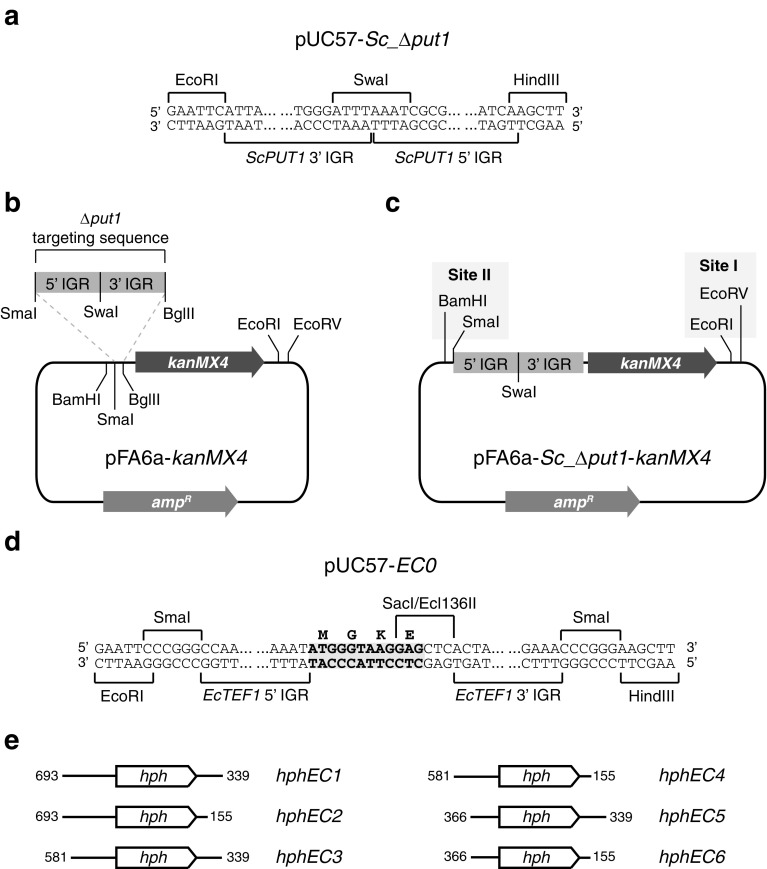
Plasmid design. **a** Design of the *S. cerevisiae PUT1* targeting cassette (*Sc_Δput1*) as inserted into the EcoRI/HindIII site of the pUC57 plasmid. **b** *Sc_Δput1* targeting cassette was inserted into the pFA6a-*kanMX4* plasmid in its reverse orientation. **c** Resulting pFA6a-*Sc_Δput1*-*kanMX4* plasmid with insertion sites I and II highlighted in grey. Plasmid components are not drawn to scale. **d** Design of the *E. cymbalariae TEF1* expression cassette (*EC0*) as inserted into the EcoRI/HindIII site of the pUC57 plasmid. The first four codons of the *EcTEF1* ORF are highlighted in grey. **e** Schematic representation of the six *hphEC* cassettes assayed in this study. The lengths (bp) of 5′ and 3′ IGRs are indicated and drawn to scale. The *hph* coding sequence is not drawn to scale

PCR primers used in this study are listed in Table 1. An *ScPUT1* targeting sequence (“*Sc_Δput1*”) consisting of two fused 200-bp segments of the *ScPUT1* 5′ and 3′ IGRs (GenBank accession AEHG01000269, residues 13,597–13,796 and 15,710–15,909, respectively) was synthesized de novo by GenScript (NJ, USA) and inserted into EcoRI/HindIII-cut pUC57 (GenBank accession Y14837) to produce the plasmid pUC57-*Sc_Δput1* (Fig. 1a). The *Sc_Δput1* sequence was amplified from pUC57-*Sc_Δput1* using primers *ScPUT1* 3′ fwd and *ScPUT1* 5′ rev. The *Sc_Δput1* amplification product was cut with BglII and SmaI and inserted in the reverse orientation into BglII/SmaI-cut pFA6a-*kanMX4* (GenBank accession AJ002680; Wach et al. 1994) to produce the plasmid pFA6a-*Sc_Δput1*-*kanMX4* (Fig. 1b, c).

**Table 1:** Primers used in this study

Primer name	Sequence (5′ → 3′)
*ScPUT1* 3′ fwd	GCG CGC AGA TCT ATT ATC AAC TCT TAT GCA CAA G
*ScPUT1* 5′ rev	GCG CGC CCG GGT GAT AAG GGA AAT AGC GCC AC
*ScPUT1* 5′ ctrl rev	TGT TCC GAT CAG CAT TAC ATG
*ScPUT1* ctrl rev	GAT GCT GTG AGA TCT GAT AAT GG
*ScPUT1* 3′ ctrl fwd	ATT TCA TCA TCC TGA GTA GCA GTA
pFA6 ctrl fwd	ACT GAG AGT GCA CCA TAT GGA
*hph* fwd	GCG CGC GAG CTC AAA AAG CCT GAA CTC ACC GCG A
*hph* rev	GCG CGC GAG CTC ATT CCT TTG CCC TCG GAC GAG T
*EcTEF1* 5′ 693 fwd	GCG CGC AAT TGC CTA TCG CTG ATC CTC ATG TA
*EcTEF1* 5′ 581 fwd	GCG CGC AAT TGA CAT GCC GCA CAC AGC ACA GA
*EcTEF1* 5′ 366 fwd	GCG CGC AAT TGT ATG CGC AGA TTG TGT ATC T
*EcTEF1* 5′ 366 fwd2	GCG CGC GGA TCC GTA TGC GCA GAT TGT GTA TCT
*EcTEF1* 3′ 339 rev	GCG CGC GAT ATC TAA TTG TAA ACT TCA TGA CT
*EcTEF1* 3′ 155 rev	GCG CGC GAT ATC CTG AGA TAT GCG CTC CTA CT

A fusion of the full-length 5′ and 3′ IGRs of *EcTEF1* gene (GenBank accession NC_016451, reverse complement of residues 841,757–842,469 and 840,021–840,379, respectively) was synthesized de novo by GenScript (NJ, USA) and inserted into EcoRI/HindIII-cut pUC57 to produce the plasmid pUC57-*EC0* (Fig. 1d). The *EC0* cassette also includes the first 12 basepairs of the *EcTEF1* open reading frame (ORF) corresponding to the amino acid sequence MGKE. The *hph* coding sequence (GenBank accession K01193) was amplified from the plasmid pFA6a-*hphNT1* (Janke et al. 2004) using primers *hph* fwd and *hph* rev. The *hph* amplification product was cut with Ecl136II and inserted into Ecl136II-cut pUC57-*EC0* to produce the plasmid pUC57-*hphEC0*.

Six *hphEC* variants (*hphEC1*-*hphEC6*, Fig. 1e) were generated by PCR amplification of pUC57-*hphEC0* using all six possible pair-wise combinations of forward primers *EcTEF1* 5′ 693 fwd, *EcTEF1* 5′ 581 fwd and *EcTEF1* 5′ 366 fwd with reverse primers *EcTEF1* 3′ 339 rev and *EcTEF1* 3′ 155 rev. Each *hphEC* variant (*hphEC1* through *hphEC6*) was cut with MfeI and EcoRV and inserted into EcoRI/EcoRV-cut pFA6a-*Sc_Δput1*-*kanMX4* (site I in Fig. 1c) to produce plasmids pFA6a-*Sc_Δput1*-*kanMX4*-*hphEC1* through -*hphEC6*.

An “expression cassette-only” control plasmid (pFA6a-*Sc_Δput1*-*kanMX4*-*EC1*) was generated by first amplifying the 5′ and 3′ *EcTEF1* IGRs from the pUC57-*EC0* plasmid using primers *EcTEF1* 5′ 693 fwd and *EcTEF1* 3′ 339 rev. The resulting *EC1* amplification product was then digested with MfeI and EcoRV and inserted into EcoRI/EcoRV-cut pFA6a-*Sc_Δput1*-*kanMX4*.

An integration plasmid where the *kanMX4* marker had been replaced by the *hphEC6* marker (pFA6a-*Sc_Δput1*-*hphEC6*) was generated by first excising the *kanMX4* marker from the pFA6a-*Sc_Δput1*-*kanMX4* plasmid through digestion with BglII and EcoRV. The *hphEC6* cassette was amplified from the pUC57-*hphEC0* plasmid with primers *EcTEF1* 5′ 366 fwd2 and *EcTEF1* 3′ 155 rev. The *hphEC6* amplification product was then digested with BamHI and EcoRV and ligated to the BamHI/EcoRV-cut pFA6a-*Sc_Δput1* fragment.

An alternative *kanMX4*/*hphEC6* integration plasmid (pFA6a-*hphEC6*-*Sc_Δput1*-*kanMX4*) was generated by amplifying the *hphEC6* cassette from the pUC57-*hphEC0* plasmid with primers *EcTEF1* 5′ 366 fwd2 and *EcTEF1* 3′ 155 rev, which was then digested with BamHI and EcoRV and inserted into BamHI/SmaI-cut pFA6a-*Sc_Δput1*-*kanMX4* (site II in Fig. 1c).

**Fig. 2:**
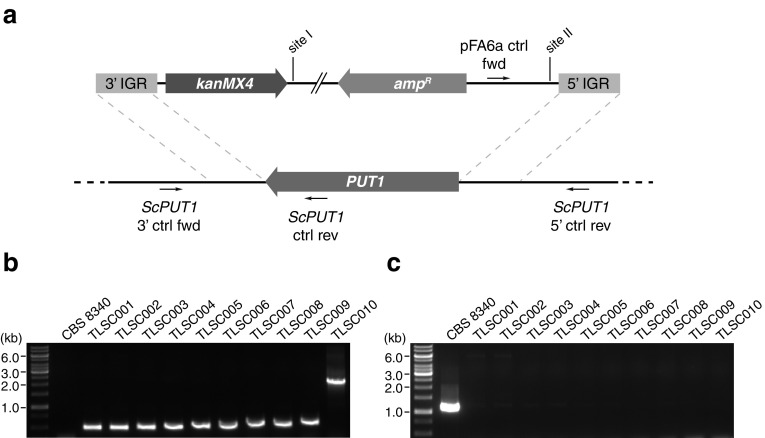
Integration of plasmid constructs at the *S. cerevisiae PUT1* locus. **a** pFA6a-*Sc_Δput1*-*kanMX4* plasmid was linearized by digestion with SwaI to enable homologous recombination with *PUT1* 5′ and 3′ IGRs. The locations of control primers to confirm correct integration of the construct are indicated. DNA elements are not drawn to scale. **b** Confirmation of the correct integration of constructs as demonstrated by PCR of genomic DNA using primers pFA6a fwd and *ScPUT1* 5′ ctrl rev. **c** Confirmation of the removal of the endogenous *PUT1* locus as demonstrated by PCR of genomic DNA using primers *ScPUT1* 3′ fwd and *ScPUT1* ctrl rev

Prior to transformation, integration plasmids were digested with SwaI to produce linearized integration constructs (Fig. 2a), which were then purified into sterile water using the QIAquick PCR purification kit (Qiagen).

### Yeast transformation

The transgenic yeast strains generated in this study are listed in Table 2. The parent yeast strain for all transformations in this study was *S. cerevisiae* CBS 8340 (syn. CEN.PK 113-7D; genotype *MATa MAL2*-*8*^*c*^ *SUC2*), which was purchased from Centraalbureau voor Schimmelcultures (Utrecht, The Netherlands). The selection agents G418 disulfate and hygromycin B were purchased from Formedium Ltd (Norfolk, UK). An aqueous stock solution of hygromycin B was prepared to a final concentration of 50 g/L, sterilized by filtration and stored at 4 °C. An aqueous stock solution of G418 disulfate was prepared to a final concentration of 100 g/L, sterilized by filtration, and stored as aliquots at − 20 °C.

**Table 2:** Transgenic yeast strains used in this study

Strain number	Relevant genotype (all strains derived from *S. cerevisiae* CBS 8340)
TLSC001	*PUT1*::*kanMX4*-pFA6a
TLSC002	*PUT1*::*kanMX4*-*EC1*-pFA6a
TLSC003	*PUT1*::*kanMX4*-*hphEC1*-pFA6a
TLSC004	*PUT1*::*kanMX4*-*hphEC2*-pFA6a
TLSC005	*PUT1*::*kanMX4*-*hphEC3*-pFA6a
TLSC006	*PUT1*::*kanMX4*-*hphEC4*-pFA6a
TLSC007	*PUT1*::*kanMX4*-*hphEC5*-pFA6a
TLSC008	*PUT1*::*kanMX4*-*hphEC6*-pFA6a
TLSC009	*PUT1*::*hphEC6*-pFA6a
TLSC010	*PUT1*::*kanMX4*-pFA6a-*hphEC6*

The transformation protocol used in this study is a simplified version of the standard lithium acetate protocol. A *S. cerevisiae* pre-culture was diluted to a final OD_600_ of 0.1 in 50-mL fresh YM broth (3-g/L yeast extract, 3-g/L malt extract, 5-g/L peptone, and 10-g/L glucose) supplemented with 75-mg/L carbenicillin and incubated at 30 °C with shaking at 200 rpm until OD_600_ reached 0.5–0.6. Cells were collected by centrifugation (2500×*g*, 5 min) and resuspended in 1.5-mL sterile water. The washed cells were collected by centrifugation (6900×*g*, 3 min), the supernatant removed and the pellet resuspended in 0.4-mL 100-mM lithium acetate. The resuspended cells were incubated at 30 °C for 15 min and then divided into 50-μL aliquots. 10-μL purified SwaI-digested integration plasmid was added to each aliquot of cells, which were then incubated at 30 °C for 15 min. 0.3 mL of a 100 mM lithium acetate/40% (w/v) PEG3350 mixture was added to each sample, and mixed and incubated for a further 15 min at 30 °C. Samples were then incubated for 15 min at 42 °C followed by immediate centrifugation (6900×*g*, 3 min) and removal of the supernatant. 0.5 mL of fresh YM broth was added to each sample, which was left to stand for 5 min at room temperature before being fully resuspended. Each sample of 0.5-mL resuspended cells were transferred to a fresh tube containing 2.5-mL fresh YM broth and incubated at 30 °C for 1 h in a rotary shaker set to 200 rpm. Samples were then centrifuged (6900×*g*, 3 min) and the supernatant discarded. The cells in each sample were resuspended in 0.2-mL fresh YM broth and spread on solid YM medium (20 g/L agar) containing either 400-mg/L G418 disulfate or 200-mg/L hygromycin B as specified in the “Results and discussion” section.

Correct chromosomal integration and the deletion of the *PUT1* locus were confirmed by PCR analysis of purified genomic DNA from each strain (Fig. 2a). Successful integration at the *PUT1* locus was assayed using primers pFA6a ctrl fwd and *ScPUT1* 5′ ctrl rev, which produce no product in the CBS 8340 parent strain, a 417-bp amplification product in strains TLSC001–TLSC009, and a 1980-bp amplification product TLSC010 due to the insertion of the *hphEC6* cassette at site II (Fig. 2b). Successful deletion of the *PUT1* coding sequence was assayed using primers *ScPUT1* 3′ ctrl fwd and *ScPUT1* ctrl rev, which produce a 1063-bp amplification product in the CBS 8340 parent strain and no product in strains TLSC001–TLSC010 (Fig. 2c).

### Comparative fitness assays of deletion strains

The absolute fitness of G418 disulfate/hygromycin B-resistant strains TLSC008 and TLSC010 was compared by parallel cultivation in minimal glucose medium (MMD) broth consisting of 6.7 g/L Difco yeast nitrogen base without amino acids (Becton, Dickinson, and Company) and 20 g/L glucose. Both strains were pre-cultured in 3 mL MMD with 200 mg/L G418 disulfate and 200 mg/L hygromycin B. Pre-cultures were diluted to a final OD_600_ of 0.05 in separate 250-mL E-flasks containing 40-mL MMD broth with 200-mg/L G418 disulfate and 200-mg/L hygromycin B. Samples were incubated at 30 °C with shaking at 200 rpm and growth was monitored by measurement of OD_600_ every 24 h for 4 days. Each strain was assayed in triplicate.

The relative fitness of G418 disulfate-resistant strain TLSC001 and hygromycin B-resistant strain TLSC009 were assayed through co-cultivation of both strains in MMD broth without selection agent. Both strains were pre-cultured in 3-mL MMD containing either 200-mg/L G418 disulfate (for strain TLSC001) or 200-mg/L hygromycin B (for strain TLSC009). 1 mL of each pre-culture was harvested by centrifugation (6900×*g*, 3 min) and resuspended in an equal volume of fresh MMD broth without selection agent. Both strains were inoculated to a final OD_600_ of 0.01 each in a single 250-mL E-flask containing 40-mL MMD broth without selection agent and incubated at 30 °C with shaking at 200 rpm. Growth of each strain was monitored through enumeration by viable count on YM solid medium containing either 200 mg/L G418 disulfate (for strain TLSC001) or 200 mg/L hygromycin B (for strain TLSC009). Co-cultures were sampled immediately following inoculation as well as after 24 and 48 h after inoculation. Co-culture experiments were performed in triplicate. Viable counts at each time point was performed in triplicate and the average expressed as colony forming units (cfu)/mL.

## Results and discussion

The abundance of sequenced yeast genomes is a valuable resource for co-opting intergenic regulatory sequences for heterologous expression of transgenes in yeast. In this study, the promoter and terminator elements contained within the 5′ and 3′ IGRs of the *E. cymbalariae TEF1* gene (*EcTEF1*, systematic gene name *Ecym_3450*) were selected for the development of a new constitutive expression cassette in yeast. At the time writing, the yeast *E. cymbalariae* is the closest relative of *E. gossypii* with a sequenced genome (Wendland and Walther 2011). The *EcTEF1* 5′ and 3′ IGRs were, therefore, considered logical choices for the development of a new expression cassette considering the relatively short evolutionary distance to the well established *EgTEF1*-based *MX* cassette.

A fusion of the full-length 5′ and 3′ *EcTEF1* IGRs was synthesized de novo and inserted into the pUC57 cloning vector. The *EcTEF1* expression cassette (from now on referred to as *EC0*) also included the first four codons of the *EcTEF1* ORF corresponding to the amino acids MGKE (Fig. 1d). This differs slightly from the *EgTEF1*-derived *MX* cassette, which includes the first eight codons of the *EgTEF1* ORF corresponding to the amino acids MGKEKTHV. The rationale for including a shorter N-terminal portion of the *E. cymbalariae* Tef1 peptide sequence was to reduce the possibility of interference with the N-terminus of transgenes that could be critical to the function of the expressed protein. The *EcTEF1* 5′ and 3′ IGRs are both significantly longer than those of the *E. gossypii* ortholog. The *EcTEF1* 5′ IGR consists of 713 bp compared to 284 bp for the *EgTEF1* 5′ IGR (GenBank accession NC_005785, reverse complement of residues 58,674–58,957), while the *EcTEF1* 3′ IGR consists of 359 bp compared to 165 bp in the *EgTEF1* 3′ IGR (GenBank accession NC_005785, reverse complement of residues 57,132–57,296).

The 5′ and 3′ IGRs from *E. cymbalariae* and *E. gossypii TEF1* genes were aligned to assess their degree of sequence conservation. The majority of the *EgTEF1* 5′ IGR sequence appeared to be conserved in the *EcTEF1* 5′ IGR, while the *EcTEF1* 5′ IGR contained two longer stretches of sequence that were not conserved in the *EgTEF1* (Fig. S1a). The largest conserved sequence block (318 bp with 53% identity and six short indels comprising a total of 57 internal gap positions) between the *EcTEF1* and *EgTEF1* 5′ IGRs was located immediately upstream of the *TEF1* ORF in both species. The majority of *EgTEF1* 3′ IGR appeared to be conserved in the *EcTEF1* 3′ IGR with the majority of the conserved sequence contained within a single 156-bp sequence block (64% identity with a single internal 8-bp indel), which was located immediately downstream of the *TEF1* ORF in both species.

Both *TEF1* 5′ IGRs were inspected for the presence of known conserved regulatory elements that are commonly found in the promoter sequences of ascomycete ribosomal protein (RP) genes (Tanay et al. 2005). Of previously characterized RP gene regulatory elements, a single putative Homolo-D site (TGTGACTG) as well as an immediately adjacent putative Rap1-binding site (CRCCCRTACAT) were found to be conserved in the 5′ end of both *TEF1* 5′ IGRs (Fig. S1a). A putative ribosomal RNA processing element (RRPE) motif (AAAAATTTT) was detected in the *E. cymbalariae TEF1* 5′ IGR, which was not conserved in the *E. gossypii TEF1* 5′ IGR (Fig. S1a). The 6-bp core sequence AATTTT of the RRPE motif is also associated with the regulation of genes required for rapid growth in budding yeasts (Ihmels et al. 2005) and is referred to as the rapid growth element (RGE). A putative RGE motif was detected in the *E. cymbalariae TEF1* 5′ IGR located just upstream of the putative RRPE/RGE motif. This RGE site was not conserved in the *E. gossypii TEF1* 5′ IGR either. Further inspection of the full-length *E. gossypii TEF1* 5′ IGR sequence identified a single putative RGE motif in the reverse orientation. However, this site overlapped with a conserved region in the *E. cymbalariae TEF1* 5′ IGR sequence, which may indicate that it is not a de facto regulatory element.

It is possible that the 318-bp conserved sequence block just immediately upstream of the *TEF1* ORF in both species may contain novel regulatory elements important for the proper expression of the *TEF1* gene. The genus *Eremothecium* belongs to the family *Saccharomycetaceae*, which includes several species with sequenced genomes such as *S. cerevisiae*, lactose-fermenting biotechnology yeasts of the genus *Kluyveromyces,* and osmotolerant spoilage yeasts of the genus *Zygosaccharomyces* among others. The abundance of available genomic sequences from species belonging to the *Saccharomycetaceae* enables the mining of genomic data for the presence of novel conserved motifs in the *TEF1* gene. However, the *TEF1* gene is found in two genomic contexts within the *Saccharomycetaceae*. In species belonging to the genera *Eremothecium*, *Kluyveromyces*, *Lachancea*, *Torulaspora,* and *Zygosaccharomyces,* the *TEF1* gene is arranged in a tandem orientation with its neighboring upstream and downstream genes *MRL1* and *MUD1*, respectively (Fig. S1b). Conversely, the *TEF1* gene is present in two near-identical paralogous copies (*TEF1* and *TEF2*) in the genera *Kazachstania*, *Naumovozyma*, *Saccharomyces*, *Tetrapisispora,* and *Vanderwaltozyma*. These genera constitute a monophyletic group originating from an ancient whole-genome duplication (WGD) event (Kellis et al. 2004). The WGD group *TEF1* paralog has retained the ancestral *MRL1*-*TEF1* IGR, while the *TEF2* paralog has retained the ancestral *TEF1*-*MUD1* IGR (Fig. S1c).

To scan for conserved sequence motifs in the *TEF1* 5′ IGR among the *Saccharomycetaceae*, the ancestral *MRL1*-*TEF1* IGR (with the *MRL1* and *TEF1* genes in a tandem orientation) must first be distinguished from the more recently derived *TKL2*-*TEF2* IGR found within the WGD group (with the *TKL2* and *TEF2* genes in a divergent orientation). In the latter case, the *TKL2* and *TEF2* genes share a common bidirectional promoter, which could introduce unwanted noise into the motif elucidation algorithm. Consequently, all *TKL2*-*TEF2* IGR sequences from WGD species of the *Saccharomycetaceae* were excluded from the further analysis in the current study. *MRL1*-*TEF1* IGR sequences from 24 species belonging to the *Saccharomycetaceae* (*Eremothecium coryli*, *E. cymbalariae*, *E. gossypii*, *Kazachstania africana*, *Kazachstania naganishii*, *Kluyveromyces aestuarii*, *Kluyveromyces lactis*, *Kluyveromyces marxianus*, *Kluyveromyces wickerhamii*, *Lachancea kluyveri*, *Lachancea lanzarotensis*, *Lachancea quebecensis*, *Lachancea thermotolerans*, *Lachancea waltii*, *Naumovozyma castellii*, *Naumovozyma dairenensis*, *S. cerevisiae*, *Saccharomyces eubayanus*, *Saccharomyces paradoxus*, *Tetrapisispora blattae*, *Tetrapisispora phaffii*, *Torulaspora delbrueckii*, *Zygosaccharomyces bailii*, and *Zygosaccharomyces rouxii*) were assayed for the overrepresentation of sequence motifs using MEME (http://meme-suite.org/tools/meme; Bailey et al. 2009). The only significant hits were the previously identified RRPE motif, which was detected in 19 out the 24 input sequences, and the shorter RGE motif, which was found in all 24 sequences. Putative Homolo-D and Rap1 sites were only found in a smaller sub-set of the sequences. No novel sites above the significance threshold (*E* = 10^−3^) were identified.

The greater lengths of the 5′ and 3′ IGRs of the *EcTEF1* gene as compared to those of the *EgTEF1* gene may be problematic for some applications: for example, decreased PCR amplification efficiency of longer linear DNA constructs. Such problems could be circumvented by trimming one or both IGRs of the *EcTEF1* gene as long as sufficient functionality could be retained. To test the potential of using trimmed variants of the *EC0* cassette, the plasmid-encoded hygromycin B phosphotransferase gene *hph* from the bacterium *Escherichia coli* was selected for expression. Hygromycin B is an aminoglycoside antibiotic produced by the actinobacterium *Streptomyces hygroscopicus* (Mann and Bromer 1958), which inhibits protein synthesis in both bacteria and eukaryotes. Expression of the *hph* gene has previously been shown to confer resistance to hygromycin B in both bacteria and yeast (Gritz and Davies 1983; Kaster et al. 1984).

The *hph* coding sequence was inserted into the *EC0* cassette to generate the *hphEC0* cassette. A spectrum of *hphEC* variants (*hphEC1* through *hphEC6*) with progressively trimmed 5′ and 3′ IGR segments was subsequently generated by PCR amplification using different primer combinations (Fig. 1e, Fig. S1a). Each *hphEC* variant was then inserted into the integration construct pFA6a-*Sc*_*Δput1*-*kanMX4* (Fig. 1c), so that the *hphEC* cassette was located immediately downstream of the *kanMX4* selection marker in a tandem orientation. The *S. cerevisiae* proline oxidase-encoding *PUT1* locus was selected as the integration site for this study as the gene is only essential for cell viability when l-proline is the only available source of nitrogen (Wang and Brandriss 1986), which allows for convenient phenotypical confirmation of transformants. A control construct containing an empty *EC1* cassette (pFA6a-*Sc*_*Δput1*-*kanMX4*-*EC1*) was included to confirm that the *EcTEF1* IGRs did not confer cryptic resistance to hygromycin B.

**Fig. 3:**
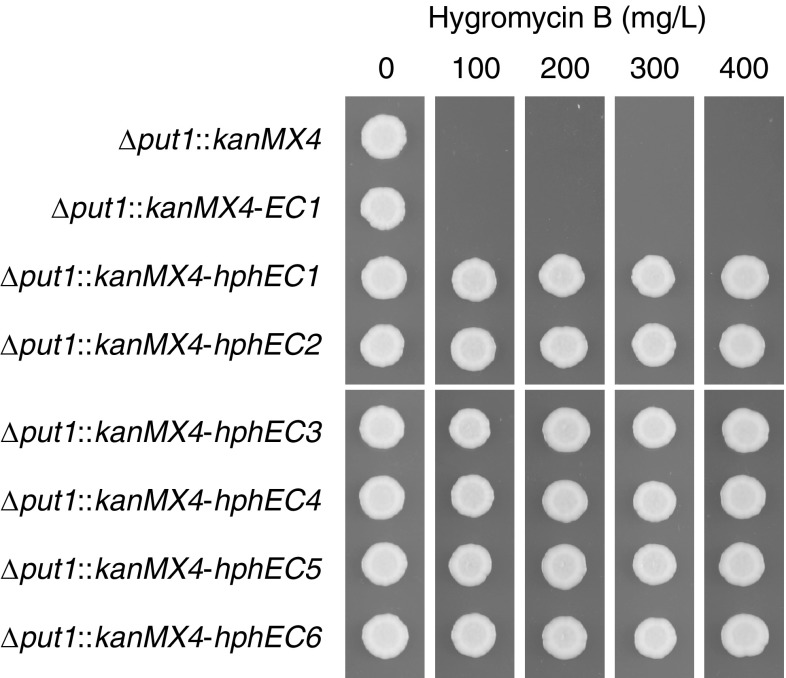
Hygromycin B tolerance of *hphEC* variants. Strains were pre-cultured overnight in 3 mL YM broth with 200 mg/L G418 disulfate and then diluted to OD_600_ 0.1 in fresh YM broth without any selection agent. 2 μL cell suspension of each strain was spotted on solid YM medium with the indicated concentration of hygromycin B. Plates were incubated for 2 days at 30 °C and then photographed

The integration constructs were used to transform *S. cerevisiae* with G418 disulfate used for selection of positive transformants. Plate growth assays of confirmed transformants containing any of the six *hphEC* variants (strains TLSC003-TLSC008) were able to grow on solid YM medium containing up to 400 mg/L hygromycin B, which was the highest concentration tested (Fig. 3). No significant growth was observed on hygromycin B-containing medium (100–400 mg/L) for strains TLSC001 and TLSC002, which had been transformed with the control constructs pFA6a-*Sc*_*Δput1*-*kanMX4* and pFA6a-*Sc*_*Δput1*-*kanMX4*-*EC1*, respectively.

The previously observed putative Homolo-D and Rap1 sites in the *EcTEF1* 5′ IGR (Fig. S1a) were shown to be dispensable for sufficient expression of the *hph* gene in *S. cerevisiae* to enable growth at hygromycin B concentrations up to 400 mg/L as demonstrated by *hphEC* variants *hphEC3* through *hphEC6*. This does not exclude the possibility that these two motifs do play a role in the proper regulation of the endogenous *TEF1* gene in *E. cymbalariae*. Another possibility is that the *MRL1* gene, which is located immediately upstream of *TEF1* in the *E. cymbalariae* genome (Fig. S1b), is the regulatory target of these sites. However, the *MRL1* gene encodes a putative membrane protein similar to mammalian mannose-6-phosphate receptors with a possible function as a sorting receptor in the delivery of vacuolar hydrolases (Whyte and Munro 2001) but does not appear to have any obvious functional connection to protein synthesis. In addition, the *E. cymbalariae MRL1* ortholog (systematic gene name *Ecym_3451*) is annotated as non-functional with a reported frame-shift mutation. The tandem orientation of the *MRL1* and *TEF1* genes would also suggest that the conserved Homolo-D and Rap1-binding sites in the *MRL1*-*TEF1* IGR, if functional, are not involved in regulating the expression of the *MRL1* gene.

All remaining experiments of the *hphEC* cassette within the current study utilized the shortest variant, *hphEC6*. The next question to be addressed was whether the adjacent *kanMX4* and *hphEC6* cassettes in the pFA6a-*Sc*_*Δput1*-*kanMX4*-*hphEC6* construct interacted in any positive manner or negative manner. For example, would the *hphEC6* selection marker retain its activity in the absence of the *kanMX4* cassette? Likewise, would the close proximity of the *kanMX4* and *hphEC6* cassettes in the pFA6a-*Sc*_*Δput1*-*kanMX4*-*hphEC6* construct cause any detectable interference between the two selection markers?

**Fig. 4:**
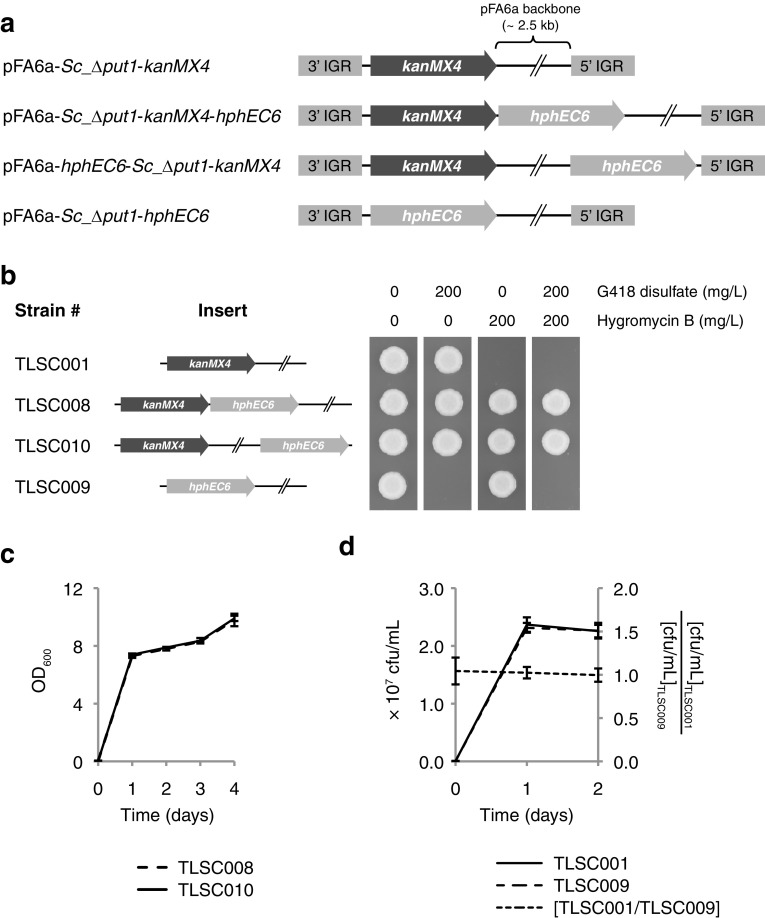
Assaying interactions and relative fitness between *kanMX4* and *hphEC6* selection markers. **a** Outline of linearized constructs used in interaction assays. Plasmid elements are not drawn to scale. **b** Tolerance of yeast strains to combinations of G418 sulfate and hygromycin B. Strains were pre-cultured overnight in 3 mL YM broth with either 200 mg/L G418 disulfate (TLSC001), 200 mg/L hygromycin B (TLSC009), or 200 mg/L of both selection agents (TLSC008 and TLSC010). Pre-cultures were diluted to OD_600_ 0.1 in fresh YM broth without any selection agent. 2 μL cell suspension of each strain was spotted on solid YM medium with the indicated concentration of G418 disulfate and hygromycin B. Plates were incubated for 2 days at 30 °C and then photographed. **c** Comparative growth dynamics in MMD broth containing both G418 disulfate and hygromycin B. Error bars indicate one standard deviation. **d** Fitness equivalence of co-cultivated *S. cerevisiae Δput1* strains carrying either the *kanMX4* or *hphEC6* marker under non-selective conditions. Error bars indicate one standard deviation

Two additional *hphEC6*-containing constructs were, therefore, generated to investigate any potential interaction between the *hphEC6* and *kanMX4* cassettes in the pFA6a-*Sc*_*Δput1*-*kanMX4*-*hphEC6* construct. The first construct replaced the *kanMX4* cassette in the pFA6a-*Sc*_*Δput1*-*kanMX4* plasmid with the *hphEC6* cassette to produce the plasmid pFA6a-*Sc*_*Δput1*-*hphEC6* (Fig. 4a). This construct would enable the evaluation of the *hphEC6* marker both as a selection marker for transformation on hygromycin B-containing medium and also allow functional study of the *hphEC6* cassette in the absence of the *kanMX4* cassette. The transformation of *S. cerevisiae* CBS 8340 was repeated with the pFA6a-*Sc*_*Δput1*-*hphEC6* construct with 200 mg/L hygromycin B as the selection agent rather than 400 mg/L G418 disulfate. Ten random colonies were picked from the selective media and screened for the presence of the pFA6a-*Sc*_*Δput1*-*hphEC6* construct at the *PUT1* locus by PCR as before (Fig. 2b, c). All ten transformants were found to have successfully integrated the pFA6a-*Sc*_*Δput1*-*hphEC6* construct and replaced the endogenous *PUT1* locus, which demonstrated the utility of the *hphEC6* as a hygromycin B-dependent selection marker. One of the transformants was selected for subsequent assays and designated strain TLSC009.

A second construct was designed where the *hphEC6* cassette was inserted at site II in pFA6a-*Sc*_*Δput1*-*kanMX4* (Fig. 1c) to make plasmid pFA6a-*hphEC6*-*Sc*_*Δput1*-*kanMX4* (Fig. 4a). Once the linearized pFA6a-*hphEC6*-*Sc*_*Δput1*-*kanMX4* construct had been integrated into the *S. cerevisiae* genome to make strain TLSC010, the *kanMX4* and *hphEC6* cassettes would be separated by the entire length of the pFA6a plasmid backbone (2479 bp). The tolerance of strains TLSC009 and TLSC010 to G418 disulfate and hygromycin B were tested using a plate growth assay and compared to strains TLSC001 and TLSC008 (Fig. 4b). As expected, strain TLSC009 was sensitive to G418 at 200 mg/L, while strain TLSC010 was resistant to both G418 disulfate and hygromycin B at 200 mg/L.

Although strains TLSC008 and TLSC010 displayed similar growth on solid medium containing both G418 disulfate and hygromycin B, the agar growth assay was only considered semi-quantitative. A growth assay in liquid medium was, therefore, performed to investigate whether the proximity between the *kanMX4* and *hphEC6* cassettes in strains TLSC008 and TLSC010 had any subtle effect of strain fitness when both resistance markers are under selection. Both strains were cultivated in parallel using a chemically defined liquid minimal medium containing both G418 disulfate and hygromycin B. The growth dynamics of both strains were monitored by optical density measurements every 24 h and shown to be both highly reproducible and indistinguishable from each other (Fig. 4c). It was, therefore, concluded that the *hphEC6* cassette could be placed immediately adjacent to the *kanMX4* cassette in a tandem orientation without any discernable effects. However, the *kanMX4* and *hphEC6* cassettes were not tested in a convergent orientation in this study and there is a possibility that the selection markers could affect the activity of each other in such an orientation.

The final question to be resolved in the present study was whether the *hphEC6* cassette was functionally equivalent to the *kanMX4* cassette in terms of strain fitness under non-selective conditions where neither selection agent is present. Relative fitness was assayed through co-cultivation of strains TLSC001 and TLSC009, which are isogenic with exception of carrying either the *kanMX4* or *hphEC6* cassette, respectively (Fig. 4a). Both strains were inoculated in chemically defined liquid minimal medium lacking any selection agent with an initial OD_600_ of 0.01 corresponding to 65,000–70,000 cfu/mL of each strain. Growth of both strains was monitored separately through enumeration by viable count on solid-rich medium containing either G418 disulfate or hygromycin B, respectively. Both strains had reached stationary phase within 24 h and no significant deviation from the initial TLSC001/TLSC009 inoculation ratio (1.04 ± 0.16) was observed at either 24 or 48 h after inoculation (Fig. 4d). It could not be excluded that significant differences in fitness between the selection markers would be observed after prolonged incubation in stationary phase. Likewise, such differences in fitness may become apparent only after repeated cycles of batch co-cultivations. However, for standard genetic applications such as heterologous expression of dominant selection markers to enable gene replacement, the *kanMX4* and *hphEC6* selection markers appear to be essentially equal in fitness.

## Conclusion

The present study has demonstrated the suitability of the *EcTEF1*-derived expression cassette *EC0* for the design of dominant selection markers in yeast engineering. The *EcTEF1* 5′ and 3′ IGRs could be trimmed down to at least 366 and 155 bp, respectively, while retaining resistance to at least 400 mg/L hygromycin B when expressing the *hph* hygromycin phosphotransferase gene (Fig. 3). Detailed expression dynamics of different variants of the *EC* cassette with regard to growth phase and nutritional status were not investigated in this study. Such information may be of importance when employing the cassette for the expression of transgenes for other purposes than dominant selection markers and would need to be determined on a case-by-case basis both in terms of transgene and strain background. Concerning the latter aspect, the differences in RP gene regulatory elements in different ascomycete fungi (Tanay et al. 2005) need to be considered as they may affect expression dynamics in a species-dependent manner.

## Electronic supplementary material

Below is the link to the electronic supplementary material.

**Fig. S1** Analysis of the *TEF1* IGRs in *E. cymbalariae* and *E. gossypii*. **a** The *EcTEF1* and *EgTEF1* 5′ and 3′ IGRs were aligned in MAFFT (https://mafft.cbrc.jp/alignment/server/). Identical sequence positions are shaded maroon. Sequence positions of the trimmed *EcTEF1* 5′ and 3 ‘IGR variants assayed in this study are indicated with arrows. Putative Homolo-D, Rap1, RRPE and RGE sites are highlighted. The *EcTEF1* coding sequence is indicated by the orange block arrow and is not drawn to scale. **b** Genomic context of the *TEF1* locus among non-WGD genera of the *Saccharomycetaceae* (*Eremothecium*, *Kluyveromyces*, *Lachancea*, *Torulaspora*, *Zygosaccharomyces*). **c** Genomic context of the *TEF1* and *TEF2* loci among WGD genera of the *Saccharomycetaceae* (*Kazachstania*, *Naumovozyma*, *Saccharomyces*, *Tetrapisispora*, *Vanderwaltozyma*). The direction of arrows indicate whether each ORF is located on the forward or reverse strand with respect to the *TEF1*/*TEF2* ORF. ORFs and IGRs are not drawn to scale. (EPS 621 kb)

## Abbreviations

*EcTEF1*: *Eremothecium cymbalariae* translational factor 1α gene
*hph*: Hygromycin phosphotransferase gene
IGR: Intergenic region
ORF: Open reading frame
RGE: Rapid growth element
RP: Ribosomal protein
RRPE: Ribosomal RNA processing element
*ScPUT1*: *Saccharomyces cerevisiae* proline oxidase gene
